# Periodontal status of maxillary central incisors after orthodontic traction: a longitudinal follow-up

**DOI:** 10.1590/1678-7757-2021-0492

**Published:** 2022-03-30

**Authors:** Louise Resti Calil, Guilherme Janson, Vinicius Merino da Silva, Marcos Roberto de Freitas, Ana Lúcia Pompéia Fraga de Almeida, Daniela Garib

**Affiliations:** 1 Universidade de São Paulo Faculdade de Odontologia de Bauru Departamento de Odontopediatria, Ortodontia e Saúde Coletiva Bauru São Paulo Brasil Universidade de São Paulo, Faculdade de Odontologia de Bauru, Departamento de Odontopediatria, Ortodontia e Saúde Coletiva, Bauru, São Paulo, Brasil.; 2 Universidade de São Paulo Faculdade de Odontologia de Bauru Departamento de Prótese e Periodontia Bauru São Paulo Brasil Universidade de São Paulo, Faculdade de Odontologia de Bauru, Departamento de Prótese e Periodontia, Bauru, São Paulo, Brasil.

**Keywords:** Tooth impacted, Incisor, Tomography X-ray computed, Traction

## Abstract

The aim of this study was to evaluate the clinical and radiographic periodontal status of impacted permanent maxillary central incisors (Mx.1) after a long term of orthodontic traction.

**Methodology:**

This split-mouth study evaluated a sample of 11 patients (five females, six males) treated with Mx.1 unilateral traction one to 28 years after the removal of orthodontic appliances. The traction Group (TG) consisted of 11 Mx.1 and the Comparison Group (CG) comprised 11 spontaneously erupted contralateral Mx.1. High-resolution CBCT exams of central incisors were performed using Accuitomo (J. Morita, Kyoto, Japan). Cross-section imagens passing through the center of maxillary central incisors were used to measure buccal and lingual alveolar bone level. Presence of fenestration, root dilacerations, root coverage, and position of the root apex were also assessed in the same images. Clinical parameters included periodontal probing depth, attachment level, gingival bleeding index, plaque index, degree of gingival recession, amount of gingival mucosa, and evaluation of interproximal papilla and black triangle. Digital model analysis included an assessment of clinical crown height and width. Intergroup comparisons were performed using paired t-, McNemar’s, and Wilcoxon tests (p<0.05).

**Results:**

Compared to CG, we found a significantly thinner labial bone plate thickness in TG at the middle (p=0.000) and apical (p=0.009) root level. We also observed an apical displaced labial bone crest level in TG (p=0.000). The Traction Group showed a greater frequency of root dilacerations and gingival recessions, a decreased amount of keratinized mucosa, and a decreased clinical attachment level at the labial aspect compared to contralateral teeth.

**Conclusions:**

A decreased thickness and height of labial alveolar bone and gingival recessions were found in maxillary central incisors 15 years after orthodontic traction. Though incisor traction might cause some periodontal impact, differences are acceptable under a clinical point of view considering the cost-benefit ratio.

## Introduction

Impaction of maxillary permanent incisors has been found in the range of 0.2-1% of the population.^1^ Moreover, the most frequent feature causing school bullying is the absence of anterior teeth.^2 , 3^ In this perspective, permanent maxillary central incisor (Mx.1) retention has a psychosocial priority to be solved. Clinically, eruption disturbances can be diagnosed when a six-month tooth eruption delay or more is observed compared to its homologue.^4 , 5^ Eruptive delays occur due to obstructive or traumatic cause.^6 - 9^ Obstructive factors involve any kind of physical barrier to the eruption, such as supernumerary teeth, tooth-bone discrepancies, gingival fibrosis, ankylosis, retained primary teeth, early loss of deciduous teeth, presence of cysts, odontomas or tumors in the region, among others.^10 - 12^ Trauma can cause germ damage or a positional change of permanent teeth, which prevents spontaneous eruption.^8^

Treatment management may include a broad range of options, including passive observation, surgical exposure and traction, and tooth extraction followed by prosthesis or lateral incisor substitution.^6 , 13 - 15^ Diagnosis and treatment planning in cases of impacted teeth require clinical and radiographic examination. Two-dimensional (2D) radiographic images were the main instrument for examination. However, these exams could normally contain distortions, positioning errors, and tooth overlaps which impaired correct analyses. CBCT (Cone Beam Computed Tomography) is the new gold standard, with a 3-dimensional (3D) parameter that enables multiple plane analyses for adequate diagnosis.^16^

Tooth traction in a closed technique is the treatment usually indicated in the literature, requiring surgical exposure, attachment placement, and orthodontic movement.^6 , 7 , 9 , 17 - 20^ However, evidence is scarce on the long-term esthetic and periodontal aspects of such cases. Three previous studies used CBCT to evaluate impacted Mx.1 treated with closed-eruption technique followed by orthodontic traction.^21 - 23^ Shi, et al. ^22^ (2015) evaluated root and alveolar bone status before and after traction and showed that, after treatment, the impacted incisor root showed the same stage of development compared to its contralateral and that both incisors had some alveolar bone loss, a thin alveolar bone surrounding the roots, and a pulp unaffected by traction. Sun, et al. ^23^ (2016) evaluated time of treatment using a CBCT taken right after orthodontic traction. The study suggested that impacted teeth treated early (stages seven or eight of Nolla method) may promote a better morphology of root apex during root development and reduce the risk of alveolar bone loss on the labial side than patients treated later (stages nine or 10 of Nolla method). Recently, Hu, et al. ^21^ (2017) analyzed the development and stability of roots and alveolar bones after orthodontic traction in impacted Mx.1. CBCT exams were taken at completion of treatment and after a two-year follow-up. In the follow-up, both the control and experimental groups showed similar root development, impacted Mx.1 had continuous and similar growth as its contralateral incisors, and roots had an increase in length and a change in direction of their apices. Additionally, neither impacted teeth nor their contralateral ones showed further alveolar bone loss during the follow-up period.

No previous study performed a longitudinal follow-up of Mx.1 after orthodontic traction analyzing CBCT. Periodontal status should be evaluated in the long-term so we can understand treatment risks and predisposing factors for gingival recessions. The aim of this study was to evaluate the periodontal status, labial and lingual alveolar bone morphology, and the periodontal clinical condition of Mx.1 at least six months after orthodontic traction. The null hypothesis was that no periodontal differences are observed for maxillary central incisors after traction, compared to its antimere.

## Methodology

### Study population

This retrospective study was approved by the Ethical Committee in Human Research at the Bauru Dental School – University of São Paulo (protocol number 1.710.788) and informed consent forms were obtained. Sample size estimation considered an alpha of 5%, a minimum difference of 3 mm to be detected, a SD of 2.32 mm for labial bone dehiscence and a statistical power of 80%.^21^ A sample size of 10 patients was required.

Orthodontic records of 1,340 patients treated in the Orthodontic Clinic at the Bauru Dental School – University of São Paulo from 1985 to 2015 were screened. Inclusion criteria for enrollment were: 1. presence of unilateral impacted Mx.1 before orthodontic treatment; 2. orthodontic traction performed during orthodontic treatment; 3. central incisor adequately leveled at the end of orthodontic treatment; and 4. debonding occurring at least six months before recruitment. Exclusion criteria were: 1. presence of craniofacial anomalies; 2. history of periodontal disease; and 3. history of gingival surgeries in the maxillary incisor region. Once patient files were selected according to inclusion criteria, patients were invited to participate and CBCTs were requested.

Overall, 18 patients met the inclusion criteria. One patient had died, three others refused to participate and three were not found. The final sample included 11 patients (five females, six male) with a mean age of 28.6 years (SD=9.32). The mean time from debonding to recruitment was 15.41 years (SD=9.48; range=1-28.3). The Traction Group (TG) consisted of 11 treated Mx.1. The Comparison Group (CG) consisted of 11 spontaneously contralateral erupted central incisors. Table 1 describes the initial position of the Mx.1 in Traction Group and Table 2 includes the ages of each patient pre-, post-treatment, and at follow-up.

**Table 1:** Etiology and position of impacted upper central incisors

Characteristics	N
**Etiology**	
Odontoma	1
Supernumerary teeth	2
**Dental trauma**	
Horizontal	2
Vertical	3
Vertically inverted	3

**Table 2:** Patient data regarding sex and age (years)

PATIENT	SEX	PRE-TREATMENT	POST-TREATMENT	FOLLOW-UP
Patient 01	MALE	8.5	11	37.2
Patient 02	MALE	12.4	14.1	34.2
Patient 03	MALE	7.3	11.7	31.2
Patient 04	MALE	9.3	13.1	31.6
Patient 05	FEMALE	10.5	16.3	21.8
Patient 06	FEMALE	11.1	12.3	15.1
Patient 07	MALE	7.8	10.2	38.6
Patient 08	MALE	9.4	17.5	38
Patient 09	FEMALE	9.1	10.7	19.3
Patient 10	FEMALE	10.5	14.6	33.7
Patient 11	FEMALE	10.7	13.1	13.5

All patients were treated with the closed eruption technique. A mucoperiosteal flap was created to expose the impacted tooth and an orthodontic button with a stainless-steel ligature was bonded to the exposed surface of the incisor using a composite adhesive system. The direction of force was vertical toward the occlusal plan using 150g for all cases. The flap was sutured leaving the ligature wire emerging in the center of the alveolar ridge. Different traction modalities were employed. Seven patients used removable appliances with coil springs; one patient received rapid maxillary expansion and a coil spring incorporated to a Haas-type expander, and three patients underwent traction with fixed appliances. During orthodontic traction, light forces were applied via elastomeric chains until the impacted tooth was exposed in the oral cavity. The attached button was removed and a bracket was bonded. The final incisor alignment was performed with 4x2 appliances or comprehensive orthodontic treatment. A Hawley retainer was used after incisor alignment in all cases. The mean duration of orthodontic traction phase was 6.63 months (SD=2.29). CBCT exams and clinical examinations were performed in the follow-up appointment.

### CBCT analyses

High-resolution CBCT exams were performed using the 3D Accuitomo (J. Morita, Kyoto, Japan) with a Field of View (FOV) of 40x40 mm and a voxel size of 0.080 mm. During the exams, patients were positioned with a parallel Frankfort plane and the sagittal plane perpendicular to the horizontal plane. DICOM files were imported into Nemoscan software (Nemotec, Madrid, Spain). Before analysis, the image position was standardized with the long axis of the central incisor coinciding with the vertical plane both in the coronal and sagittal sections.

**Figure 1:**
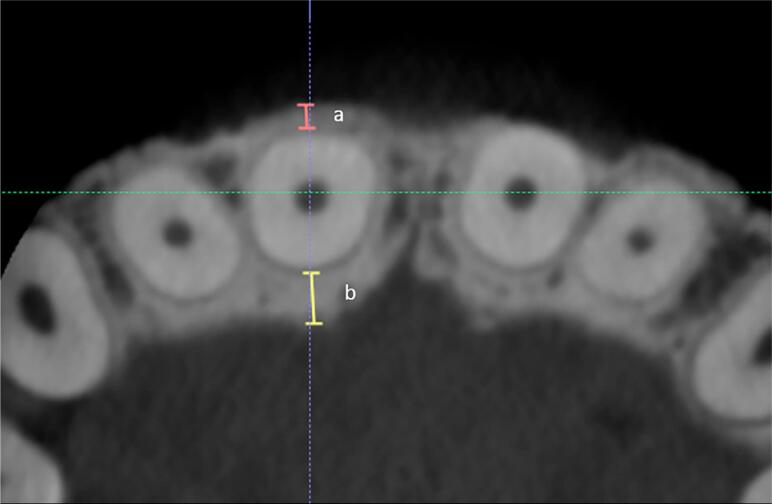
Buccal bone plate thickness (A): distance from the buccal root surface to the farthest alveolar bone surface, measured perpendicularly to the long axis of the tooth at the middle and apical third of the root; Lingual bone plate thickness (B)

**Figure 2:**
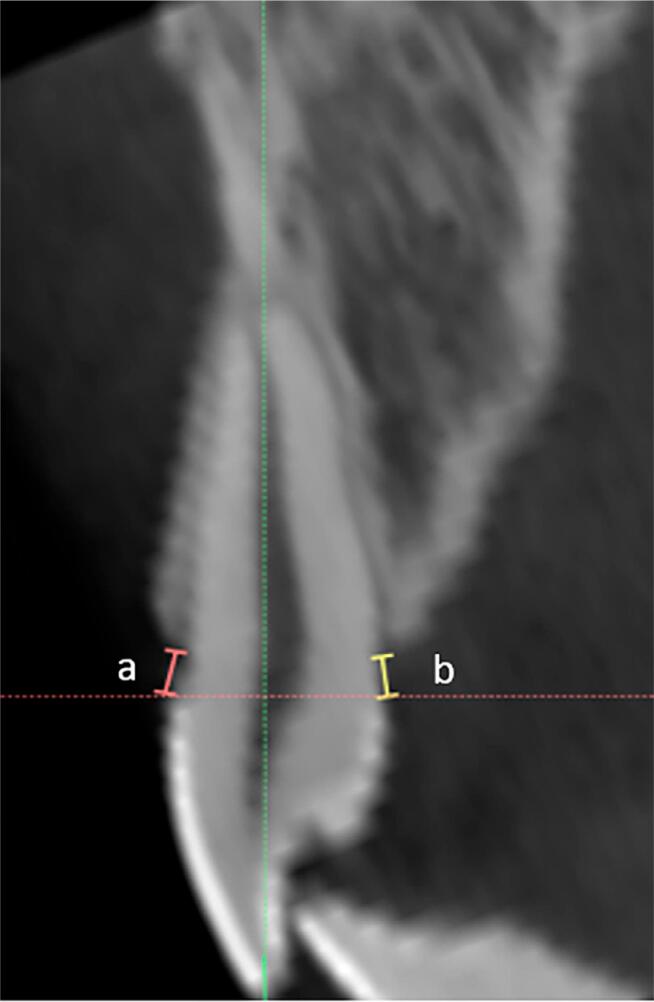
Buccal alveolar crest height (A): distance between the cemento-enamel junction and the alveolar bone crest at the most coronal level of the alveolar bone in the buccal aspect. Lingual alveolar crest height (B): distance between the cemento-enamel junction and the alveolar bone crest at the most coronal level of the alveolar bone in the lingual aspect

Labial and lingual bone plate thickness (LaBT and LBT) were measured on axial sections passing between the maxillary central incisor root thirds ( Figure 1 ). Labial and lingual alveolar crest heights were measured on cross sections passing through the center of the root canal of each central incisor ( Figure 2 ). Using the same cross sections, the following parameters were also analyzed: presence of labial bone fenestration; percentage of labial bone height (0%, 25%, 50%, 75%, and 100% as categorical data) on the labial and lingual aspects of incisor roots; presence or absence of root dilacerations; and the position of the root apex as centered (C), labially (La) or lingually (L) displaced.

### Clinical measurements

In both groups, periodontal clinical examination was performed using a Williams (Hu-friedy, USA) periodontal probe. Gingival recession (GR), probing depth (PD), clinical attachment level (CAL), gingival bleeding index (B), plaque index (P), amount of keratinized mucosa (KM), and presence of interdental black space (BT) were recorded.^24 , 25^

All periodontal clinical measurements were performed at six sites including the mesial, center, and distal regions of labial and lingual crown surfaces. Identification of the mucogingival junction was performed using Schiller’s IKI solution staining.^26^

To evaluate plaque index (P), all teeth were air-dried and examined with a plaque disclosing agent (0.6% malachite green solution prepared by the Biochemistry Department). Presence or absence of plaque was evaluated in a binomial pattern in which visible plaque received grade 1 and absence of plaque received grade 0. Presence of interdental black spaces (BT) on the mesial or distal aspects of each maxillary central incisor was recorded.

### Digital models

Conventional dental models were obtained after clinical evaluation. Dental models were scanned using a 3Shape R700 3D scanner (3Shape A/S, Copenhagen, Denmark). Clinical crown height was measured from the gingival margin to the incisal edge according to Andrews. Also, the width of both central incisors were evaluated using the OrthoAnalyzerTM 3D software (3Shape A/S, Copenhagen, Denmark).^27 , 28^

### Error study

CBCT and dental models were measured twice by the same examiner within a month’s interval. Random and systematic errors were estimated by comparing the first and second measurement with the Dahlberg formula and paired t-tests (p<5%), respectively.

### Statistical Analyses

The Kolmogorov-Smirnov test showed normal distribution of the labial and lingual variables (middle and apical bone plate thickness, alveolar crest height, dental crown height and width, height/width ratio, gingival recession, probing depth, clinical attachment level, gingival bleeding index, plaque index, and amount of keratinized mucosa). Intergroup comparisons of all variables were performed using paired t-tests. For qualitative intergroup analysis, the McNemar’s (presence of dilaceration, presence of fenestration, position of the root apex) and Wilcoxon tests (labial bone height) were used. The Pearson correlation coefficient was used to evaluate the relation between CBCT images and clinical findings. Results were considered at p<0.05. The statistical analyses were performed using the Statistica software (version 10.0; Statsoft, Tulsa, Okla).

## Results

Random errors for measurements performed on CBCT exams and digital dental models varied from 0.23 (lingual bone plate thickness in the apical third) to 0.35 mm (labial alveolar crest height). No significant systematic error was found.

Maxillary central incisors showed a significantly thinner labial bone plate in the middle and apical root levels in the Traction Group with a difference of approximately 0.5 mm ( Table 3 ). A decreased labial alveolar crest height was observed in TG compared to CG with a mean difference of 2.3 mm ( Table 3 ). The Traction Group showed greater gingival recession (difference of 0.45 mm), decreased amount of keratinized mucosa (difference of 0.9 mm), and a more apically displaced attachment level (difference of 0.66 mm) than the CG ( Table 3 ).

**Table 3:** Intergroup comparison of quantitative variables, gingival bleeding, and plaque index (Paired t–test)

n=11 Variables	TG Mean (SD)	CG Mean (SD)	Difference	95% CI Lower, Upper	p
Labial bone plate thickness (middle) (mm)	0.10 (0.26)	0.67 (0.30)	-0.56	-0.82. -0.31	0.000 *
Labial bone plate thickness (apical) (mm)	0.34 (0.34)	0.80 (0.22)	-0.46	-0.72. -0.20	0.009 *
Lingual bone plate thickness (middle) (mm)	1.23 (1.11)	1.07 (0.49)	0.68	-0.61. 0.91	0.549
Lingual bone plate thickness (apical) (mm)	2.48 (2.33)	3.23 (1.60)	-0.74	-2.52. 1.04	0.216
Labial alveolar crest height (mm)	4.78 (1.59)	2.42 (0.99)	2.36	1.18. 3.54	0.000 *
Lingual alveolar crest height (mm)	1.92 (1.12)	1.34 (0.51)	0.57	-0.20. 1.35	0.133
Dental crown height (mm)	10.57 (1.65)	9.94 (1.69)	0.62	-0.86. 2.11	0.114
Gingival recession (labial) (mm)	0.60 (0.51)	0.15 (0.31)	1.18	0.37. 2.00	0.016 *
Gingival recession (lingual) (mm)	0.09 (0.21)	0.00 (0.00)	0.79	0.01. 1.57	0.193
Probing Depth (labial) (mm)	2.24 (0.39)	1.96 (0.48)	0.86	-0.11. 1.84	0.158
Probing Depth (lingual) (mm)	2.27 (0.35)	2.18 (0.27)	0.94	0.10. 1.79	0.518
Clinical attachment level (labial) (mm)	2.66 (0.51)	2.00 (0.44)	0.87	0.00. 1.73	0.022 *
Clinical attachment level (lingual) (mm)	2.36 (0.48)	2.15 (0.27)	0.89	0.00. 1.78	0.224
Amount of keratinized mucosa (mm)	5.27 (1.79)	6.18 (1.66)	-0.9	-2.44. 0.62	0.033 *
Gingival bleeding index (labial)	0.45 (0.47)	0.45 (0.48)	0.28	-0.34. 0.91	1.000
Gingival bleeding index (lingual)	0.39 (0.46)	0.42 (0.49)	0.09	-0.60. 0.78	0.755
Plaque index	0.47 (0.50)	0.36 (0.50)	0.09	-0.36. 0.54	0.242

* Statistically significant (p<0.05)

The experimental group showed significantly greater frequency of root dilacerations (72.7%) and smaller bone coverage on the root labial aspect compared to the CG ( Table 4 ). Black spaces were not found in both groups. A moderately inverse correlation (r=-0.64) was found between labial bone plate thickness and attachment level in the Traction Group ( Table 5 ). A moderately positive correlation (r=0.59) was found between labial alveolar crest height and attachment level ( Table 5 ).

**Table 4:** Intergroup comparison of the frequency (%) of qualitative periodontal parameters (McNemar’s test^‡^ and Wilcoxon test^†^)

Presence	TG	CG	p
Presence of dilaceration	8 (72.7%)	0 (0%)	0.013 *^‡^
Presence of fenestration	1 (9.1%)	0 (0%)	1.000 ^‡^
Labial alveolar bone height	50%	100%	0.007 *^†^
Lingual alveolar bone height	100%	100%	0.067
Presence of black space	0%	0%	
**Position of the root apex**				
Labial	8 (72.7%)	10 (90.9%)	0.083^‡^
Center	2 (18.2%)	1 (9.1%)	
Lingual	1 (9.1%)	0 (0%)	

* Statistically significant

**Table 5:** Correlation between clinical and CBCT findings (Pearson correlation coefficient)

CBCT variables and clinical parameters	r	p
Labial bone plate thickness (middle) vs probing depth (labial)	-0.114	0.613
Lingual bone plate thickness (middle) vs probing depth (lingual)	-0.201	0.37
Labial bone plate thickness (middle) vs clinical crown height	-0.477	0.025*
Labial alveolar crest height vs clinical crown height	0.455	0.034*
Lingual alveolar crest height vs clinical crown height	-0.081	0.721
Labial alveolar crest height vs probing depth (labial)	-0.018	0.936
Lingual alveolar crest height vs probing depth (lingual)	0.251	0.26
Labial bone plate thickness (middle) vs recession	-0.205	0.36
Labial bone plate thickness (middle) vs amount of keratinized mucosa	0.357	0.102
Labial bone plate thickness (middle) vs attachment level	-0.646	0.001*
Labial alveolar crest height vs recession	0.141	0.532
Labial alveolar crest height vs amount of keratinized mucosa	-0.443	0.039*
Labial alveolar crest height vs attachment level	0.597	0.003*

## Discussion

This is the first study to analyze the periodontal status of Mx.1 15 years, on average, after treatment correlating CBCT outcomes and periodontal clinical findings. Most previous studies on Mx.1 were clinical reports, clinical periodontal evaluations or radiographic assessments. Only three recent studies evaluated treatment of Mx.1 by means of three-dimensional images, but their maximum post-treatment evaluation time was two years.^21 - 23^ Variability in the initial position of impacted incisors and diverse traction mechanics are limitations of our study. Three previous CBCT studies collected a homogeneous sample with inverted impacted maxillary incisors. However, the long-term nature of our study restricted exclusions due to initial incisor positions.

Computed tomography is the only current imaging method that enables visualization of buccal/labial and lingual bone plates.^29^ With high image definition and high sensitivity, CBCT images can reveal bone dehiscence and fenestrations.^30 - 33^ On the other hand, limitations are found for dehiscence identification. Variations in image acquisition settings, including field of view and voxel size, influence submillimeter accuracy.^34^ A false-positive diagnosis for bone dehiscence and an overestimation of crest level can occur when very thin bone plates are present.^32 , 35^ However, a small voxel size and small field of view was used in our study, contributing to a small study error.

Results showed that, on average, 15 years later there was not a significant labial bone loss in the analyzed sample. TG showed both thinner labial alveolar bones and greater labial bone dehiscence than spontaneously erupted contralateral incisors ( Table 3 ). Labial bone loss is a common complication of orthodontic traction, as shown in previous studies using high-resolution CBCT.^22 , 23^ A previous study reported that impacted maxillary incisors showed reduced buccal bone height after treatment and that buccal bone loss is discontinued and remained stable two years after treatment.^21^ In the sample, most of the impacted central incisors showed malposition before traction and, therefore, were more prone to labial bone dehiscence after traction. Surgical management of an impacted tooth is also a possible explanation for labial bone loss.^19^ Several surgical techniques are adequate for orthodontic traction.^6 , 7 , 9 , 17 - 20^ The technique that was used in this study was widely indicated in the literature, requiring surgical exposure, attachment placement, and closed eruption.^6 , 7 , 9 , 17 - 20^ During the exposure technique, a conservative removal of the surrounding bone to bond the traction attachment is necessary. Traction direction should also be controlled to move the incisors toward the center of the alveolar bone crest ^36 , 37^ . On the other hand, the lingual alveolar bone was similar in both groups, in accordance with previous studies.^21 - 23^

Labial bone dehiscence is a risk factor for the development of gingival recessions.^13 , 38 , 39^ TG showed more gingival recession on the labial aspect than CG ( Table 3 ). The amount of gingiva was also smaller in the Traction Group compared to its antimere ( Table 3 ). In this study, the mean gingival recession in the labial aspect found between the two groups was 0.45 mm. A previous study found gingival recessions of 0.21 mm in maxillary incisors after orthodontic traction using a closed eruption technique.^40^ Previous studies also reported that gingival recessions are often observed after orthodontic traction of maxillary central incisors.^13 , 41^ This sample also showed a 73% ratio of gingival resection in TG and no correspondent answer was found in CG.

On the labial aspect, the Traction Group also showed an apically displaced attachment level compared to CG ( Table 3 ). This result is in agreement with a previous study showing a decreased attached gingiva in patients treated with closed-eruption incisors.^20^ Loss of attachment may be associated to orthodontic procedures and toothbrushing injuries.^42^ In this study, attachment level was correlated with a thinner labial bone plate and with an increased labial alveolar crest height ( Table 5 ). Labial dehiscence is a predisposing factor for loss of attachment.^39 , 43^ The probing depth in TG was similar to CG. These findings are in agreement with a previous study and are probably explained by the development of a long connective attachment replacing labial bone loss.^20 , 39^ No intergroup difference was found for gingival bleeding index, and clinical crown height and width, and no black spaces were found in both groups.

The Traction Group showed a greater frequency of root dilacerations than its antimeres ( Table 4 ). These findings are explained by trauma as the main etiological factor of incisor impaction in our sample ( Table 1 ). Although root dilacerations were present, most of the root apex were within the limits of the alveolar ridge ( Table 4 ). Previous studies analyzing impacted inverted maxillary central incisors also found many root dilacerations, Shi, et al.^22^ (2015) found 20 dilacerations out of 30 impacted central incisors and Sun, et al.^23^ (2016) showed a 50-95% of root dilaceration. When impacted central incisors with root dilacerations are treated early, roots continued to develop and the severity of the dilacerations decreased.^22 , 23^ Dilaceration is the probable explanation for variations observed in the labiolingual apex position in the Traction Group ( Table 4 ). The 2x4 mechanics align incisors using clinical parameters of crown positioning with limitations to reach an ideal position of the root apex when root dilacerations are present.

Although orthodontic traction using a closed-eruption technique is an accepted clinical method, slight negative esthetic and periodontal effects on the treated tooth should be expected. Patients should also be informed of periodontal risks and the possible need for additional procedures at the end of orthodontic treatment, including gingival grafts. However, the benefits of traction surpass the side-effects of the therapy.

Limitations of this study include the wide range of follow-up time after debonding when the assessment was performed. However, differences in follow-up time would affect both groups. Another limitation was that the patients were treated by different orthodontists with different anchorage units used during traction. However, the frequency of impacted maxillary central incisors is very low, requiring the screening of thirty years of clinical records to select the adequate sample size. Additionally, these variations are expected in retrospective studies. Further studies comparing long-term smile esthetic assessment by professionals, laypersons, and patient self-perception should be performed.

## Conclusion

The null hypothesis was rejected. The long-term periodontal condition of maxillary central incisors after orthodontic traction was distinct, compared to its antimere. A decreased thickness and height of labial alveolar bone and a greater number of gingival recessions were observed in maxillary central incisors long-term after orthodontic traction. Though incisor traction might cause some periodontal impact, differences are acceptable under a clinical point of view considering its cost-benefit ratio.
